# Eruptive Keloids in a Young Female Following Varicella: A Case Report

**DOI:** 10.1002/ccr3.72579

**Published:** 2026-04-19

**Authors:** Sandesh Shah, Rabin Baniya, Joshana Shrestha, Mohan Bhusal, Deepika Neupane, Radhika Maharjan

**Affiliations:** ^1^ Department of Dermatology Nepal Medical College and Teaching Hospital Kathmandu Nepal; ^2^ Department of Prosthodontics B.P. Koirala Institute of Health Sciences Dharan Nepal

**Keywords:** chickenpox, eruptive keloid, pediatric dermatology, scar formation, varicella

## Abstract

Keloids are fibroproliferative scars extending beyond the original wound margin and are more common in individuals with darker skin types. Eruptive keloids represent a rare clinical presentation characterized by the sudden development of multiple lesions. Only a few cases have been reported following varicella infection. We report a 15‐year‐old immunocompetent female who developed five painful and pruritic keloids over the jaw, chest, abdomen, and flank within 2 months of primary varicella infection. All lesions corresponded to healed varicella scars. Diagnosis was made clinically based on classical morphology. The patient was managed conservatively due to financial constraints, and lesions remained stable during follow‐up. Varicella‐induced inflammatory and profibrotic cytokine responses may contribute to aberrant wound healing. Clinicians should recognize this rare temporal association to ensure early counseling and appropriate management.

## Key Clinical Message

Varicella infection may rarely precede the formation of eruptive keloids in immunocompetent adolescents. Early recognition, counseling, and timely multimodal management are essential to minimize long‐term physical and psychosocial morbidity.

## Graphical abstract

Development of multiple eruptive keloids in a 15‐year‐old female following varicella infection, highlighting a rare temporal association and the role of inflammatory and profibrotic pathways in aberrant wound healing.

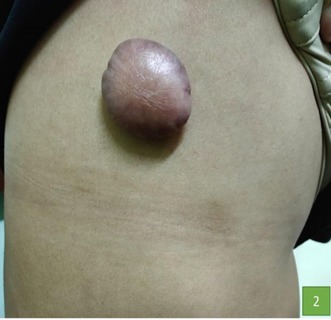

## 1 Introduction

Keloids are pathological scars characterized by fibroblast hyperproliferation and excessive collagen deposition that extends beyond the original wound margin. They affect approximately 5%–16% of the population, with higher prevalence in individuals with darker skin types [1]. Eruptive keloids are defined as the sudden appearance of multiple keloidal lesions within a short period, typically within 6 months, in the absence of significant preceding trauma [2]. Only five cases of varicella‐associated eruptive keloids have been reported worldwide (Table 1) [2, 3, 4, 5, 6, 7]. A structured search of PubMed, Scopus, and Google Scholar (1940–2024) confirmed these reports.

**TABLE 1:** Comparison of varicella‐associated eruptive keloid cases.

Case (year)	Age/sex	Number of keloids	Onset after varicella	Distribution	Histology	Treatment	Outcome
Traub (1944)	12/F	Multiple	3–4 weeks	Chest, back	No	None	Stable
Thomas (1946)	15/M	> 10	1–2 months	Trunk, arms	No	None	Persistent
Scheinfeld (2000)	5/M	Multiple	1 month	Trunk, face	No	None	Stable
Kluger (2011)	17/M	Numerous	6 months	Back, shoulders	No	Corticosteroids	Partial response
Deepika (2014)	14/F	Multiple	2–3 months	Trunk	No	Symptomatic care	Stable
Present case (2026)	15/F	5	Within 2 months	Jaw, chest, abdomen, flank	No	Symptomatic care	Stable

*Note:* Table summarizes previously reported varicella‐associated eruptive keloid cases and highlights similarities and differences with the present case.

Varicella infection induces the release of widespread cytokines (IL‐1, IL‐6, TNF‐α), which may shift wound healing toward a profibrotic phenotype [8, 9, 10]. Profibrotic cytokines such as TGF‐β1 and PDGF amplify fibroblast proliferation and extracellular matrix deposition, which are implicated in keloid formation [11].

## 2 Case History/Examination

A 15‐year‐old previously healthy female developed multiple firm nodules over the right jaw, chest, abdomen, and right flank within 2 months of primary varicella infection, which had been diagnosed clinically and treated with acyclovir (800 mg orally, five times daily for 1 week), initiated approximately 1 day after the onset of symptoms. She had not received the varicella vaccine. No topical or systemic medications were used other than oral acyclovir.

She complained of pruritus and pain. There was no personal or family history of keloids, and she was HIV‐negative. Her Fitzpatrick skin type was IV.

**FIGURE 1:**
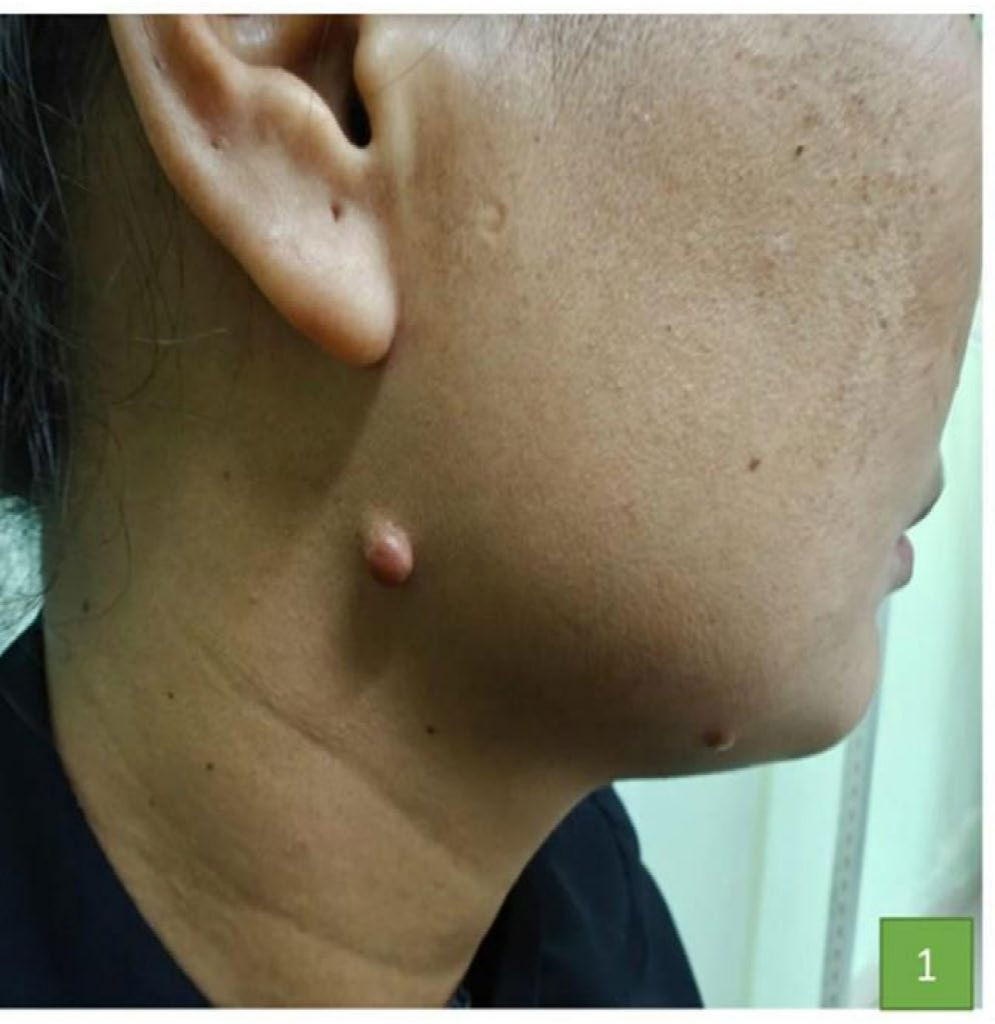
Presence of two skin‐colored nodules over the right lower jaw of size (2 × 2 cm) and (1 × 1 cm).

**FIGURE 2:**
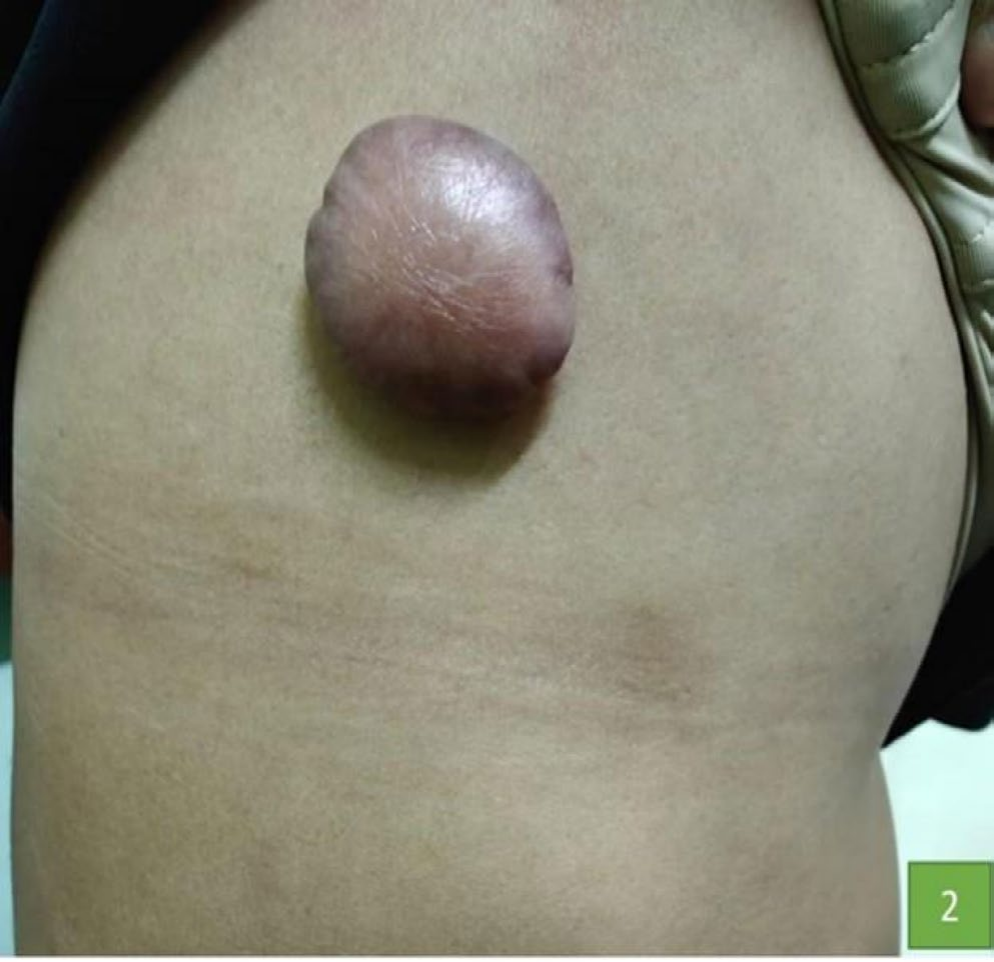
Presence of a keloid of size 4 × 4 cm over the chest.

**FIGURE 3:**
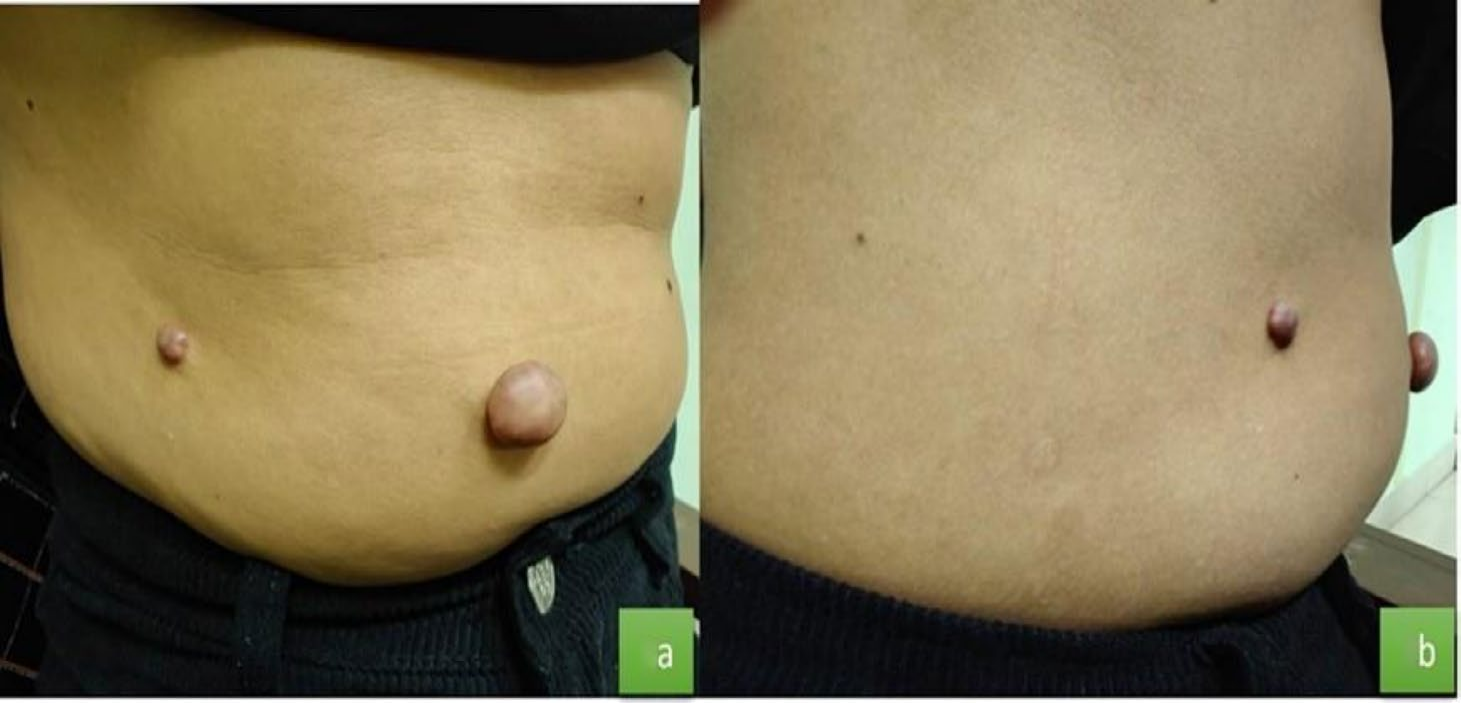
Multiple eruptive keloids over the right flank and abdomen (2 × 2 cm to 1 × 1.5 cm).

A total of 5 lesions were identified, measuring 1 × 1 cm to 4 × 4 cm, all arising over healed varicella scars (Figures 1, 2, 3).

## 3 Investigation and Treatment

Although histopathology was not performed, the lesions demonstrated classical keloid morphology, characterized by firm consistency, raised borders, smooth, shiny surface, and extension beyond original scar margins, providing high diagnostic confidence.

Management options included intralesional corticosteroids, cryotherapy, and surgical excision. Active keloid‐directed therapies were deferred due to financial limitations and patient preference. Because symptomatic lesions were stable without rapid progression, the family opted for symptomatic management only (antihistamines, paracetamol).

## 4 Follow‐Up and Outcome

The patient was followed for 3 months. Lesions remained stable in size without rapid progression.

## 5 Discussion

Varicella infection triggers the release of IL‐1, IL‐6, and TNF‐α, creating a proinflammatory environment conducive to excessive fibroproliferation [10].

Profibrotic cytokines such as TGF‐β1 and PDGF further stimulate fibroblast proliferation and collagen dysregulation, key components of keloid pathogenesis [11].

Varicella‐zoster virus establishes lifelong latency in dorsal root ganglia, and subclinical reactivation has been linked to persistent low‐grade immune activation. Previous studies have shown ongoing expression of proinflammatory cytokines, including IL‐6 and TNF‐α, even in the absence of visible clinical lesions. This persistent inflammatory environment could potentially contribute to abnormal wound healing and fibroproliferation. However, direct evidence connecting latent or subclinical VZV infection to keloid formation remains limited, and this proposed mechanism should be viewed with caution [10, 12, 13]. These mechanisms may support a possible viral contribution to aberrant scar formation [14].

Notably, only a minority of individuals develop keloids after skin injury or inflammation, indicating a role for individual susceptibility. Genetic factors, darker skin tones, and differences in immune and fibroproliferative responses are thought to influence this specific risk [1].

Differential diagnoses—including hypertrophic scars, dermatofibroma, prurigo nodularis, and sclerotic disorders—were considered. Hypertrophic scars were considered less likely because the lesions extended beyond the original wound margins. Dermatofibroma was ruled out due to the lack of a firm dermal papule with a positive dimple sign. Prurigo nodularis and sclerotic disorders were excluded based on clinical appearance, distribution, and their temporal relationship with prior varicella lesions [7, 8, 9]. In our case, the absence of prior keloids and the temporal association with varicella infection support the hypothesis that systemic immunologic dysregulation contributed to the development of eruptive lesions. Although varicella‐associated eruptive keloids are rare, similar fibroproliferative responses have been reported following other inflammatory or infectious triggers. These include herpes zoster infection and, in rare cases, vaccination‐related cutaneous inflammation [15, 16]. While the timing between varicella infection and keloid formation is notable, a causal link cannot be confirmed based on a single case.

First‐line treatment generally includes intralesional corticosteroids, often combined with adjunctive therapies such as cryotherapy or silicone gel sheeting. Refractory cases may benefit from multimodal approaches including 5‐fluorouracil, laser therapy, bleomycin, or surgical excision with adjuvant therapy to decrease recurrence [17, 18]. Equally important is psychosocial support, particularly for adolescents facing stigmatization and self‐image concerns [19]. Psychosocial assessment was conducted using an informal clinical interview; however, standardized scoring with the DLQI was planned for follow‐up. Due to resource limitations, a formal psychological referral was not completed.

Prognosis varies. Lesions usually persist, may grow larger over time, and have a high recurrence rate when treated only surgically. Possible complications include ongoing pain, pruritus, secondary infection, or psychosocial distress.

Although histopathological confirmation was not conducted due to resource limitations, the diagnosis was made with high clinical confidence based on classical morphology. Nevertheless, histopathology remains the gold standard, and its absence is a significant limitation. Furthermore, the relatively short follow‐up period restricts the ability to evaluate long‐term progression, recurrence, and treatment outcomes.

## 6 Conclusion

Varicella infection may be temporally associated with eruptive keloid development, although causality cannot be inferred from a single case. Early recognition and appropriate multimodal management are essential to optimize patient outcomes.

## Author Contributions

**Sandesh Shah:** conceptualization, methodology, project administration, resources, writing – original draft, writing – review and editing. **Rabin Baniya:** conceptualization, project administration, resources, writing – review and editing. **Joshana Shrestha:** conceptualization, methodology, project administration, resources, writing – original draft, writing – review and editing. **Mohan Bhusal:** project administration, resources, writing – review and editing. **Deepika Neupane:** project administration, resources, writing – review and editing. **Radhika Maharjan:** project administration, resources, writing – review and editing.

## Funding

The authors have nothing to report.

## Consent

Written informed consent was obtained from the patient's legal guardian in accordance with the journal's patient consent policy. I will retain the original written consent form and provide it to the Publisher if requested.

## Conflicts of Interest

The authors declare no conflicts of interest.

## Data Availability

The authors have nothing to report.
